# Causal Relationship between Adiponectin and Metabolic Traits: A Mendelian Randomization Study in a Multiethnic Population

**DOI:** 10.1371/journal.pone.0066808

**Published:** 2013-06-24

**Authors:** Andrew Mente, David Meyre, Matthew B. Lanktree, Mahyar Heydarpour, A. Darlene Davis, Ruby Miller, Hertzel Gerstein, Robert A. Hegele, Salim Yusuf, Sonia S. Anand, for the SHARE and SHARE-AP Investigators

**Affiliations:** 1 Population Health Research Institute, Hamilton, Canada; 2 Department of Clinical Epidemiology and Biostatistics, McMaster University, Hamilton, Canada; 3 Blackburn Cardiovascular Genetics Laboratory, Robarts Research Institute, London, Ontario, Canada; 4 Department of Medicine, University of Western Ontario, London, Ontario, Canada; 5 Six Nations Health Services, Ohsweken, Canada; 6 Department of Medicine, McMaster University, Hamilton, Canada; University of Colorado Denver, United States of America

## Abstract

**Background:**

Adiponectin, a secretagogue exclusively produced by adipocytes, has been associated with metabolic features, but its role in the development of the metabolic syndrome remains unclear.

**Objectives:**

We investigated the association between serum adiponectin level and metabolic traits, using both observational and genetic epidemiologic approaches in a multiethnic population assembled in Canada.

**Methods:**

Clinical data and serum adiponectin level were collected in 1,157 participants of the SHARE/SHARE-AP studies. Participants were genotyped for the functional rs266729 and rs1260326 SNPs in *ADIPOQ* and *GCKR* genes.

**Results:**

Adiponectin level was positively associated with HDL cholesterol and negatively associated with body mass index, waist-to-hip ratio, triglycerides, fasting glucose, fasting insulin, systolic and diastolic pressure (all P<0.002). The rs266729 minor G allele was associated with lower adiponectin and higher HOMA-IR (P = 0.004 and 0.003, respectively). The association between rs266729 SNP and HOMA-IR was no longer significant after adjustment for adiponectin concentration (P = 0.10). The rs266729 SNP was associated with HOMA-IR to an extent that exceeded its effect on adiponectin level (0.15 SD 95% C.I. [0.06, 0.24], P<0.001). There was no significant interaction between rs266729 SNP and ethnicity on adiponectin or HOMA-IR. In contrast, the SNP rs1260326 in *GCKR* was associated with HOMA-IR (P<0.001), but not with adiponectin level (P = 0.67).

**Conclusion:**

The association of the functional promoter polymorphism rs266729 with lower serum adiponectin and increased insulin resistance in diverse ethnic groups may suggest a causal relationship between adiponectin level and insulin resistance.

## Introduction

Adiponectin is a secretagogue exclusively produced by adipocytes and abundantly secreted into the bloodstream where it accounts for 0.01% of total plasma protein [1]. Extensive study of adiponectin function in animal models led to the conclusion that adiponectin promotes insulin sensitivity in the muscle and liver [2], [3]. Cross-sectional epidemiological studies in humans have shown that low serum adiponectin is associated with insulin resistance, obesity, dyslipidemia, coronary artery disease, hypertension or type 2 diabetes (T2D) [4]–[8]. In longitudinal studies, hypoadiponectionemia was shown to predict the development of insulin resistance [9], dyslipidemia [10], T2D [11], hypertension [12], coronary artery disease [13] but was not a predictor of subsequent weight gain [14]. Heritability studies provided strong evidence that plasma adiponectin level is genetically determined and that an overlap exists between the genetic architecture of adiponectin and features of the metabolic syndrome such as fasting insulin or HDL-cholesterol [15], [16]. The gene encoding adiponectin (*ADIPOQ*) is located on chromosome 3q27 and comprises three exons [17]. Candidate gene studies [18], and more recently genome-wide association (GWA) studies [19], fine-mapping [20] or large-scale deep resequencing experiments [21] have identified numerous common and rare variants at the *ADIPOQ* locus associated with serum adiponectin level. Even though *ADIPOQ* is the major genetic determinant of serum adiponectin level, 12 additional contributing loci have been identified through large-scale GWA meta-analyses [22]–[25]. Datsani and colleagues recently showed convincing evidence of association between a multiple SNP adiponectin-decreasing gene score and decreased body mass index (BMI), increased waist to hip ratio (WHR), higher triglyceride (TG) levels, lower HDL-C concentrations, higher post oral glucose tolerance test (OGTT) 2-hr glucose level, higher homeostatic model assessment-insulin resistance (HOMA-IR) and increased T2D risk using large datasets from international consortia [25]. Overall, these data suggest that adiponectin may be a key hormone in the development of metabolic syndrome.

The common SNP -11377 C>G (rs266729) is located in the promoter region of the gene *ADIPOQ* and has functional consequences on *ADIPOQ* gene expression [26]. The rs266729 G variant allele has been consistently associated with lower serum adiponectin concentration in diverse ethnic groups [18], [27] and with increased risk of T2D [28] and coronary artery disease [29]. In this study we investigated the causal association between serum adiponectin level and the different components of the metabolic syndrome, using both observational and genetic epidemiology approaches in a multiethnic population randomly assembled in Canada.

## Subjects and Methods

### Ethics Statement

This study was approved by the Hamilton Health Sciences/Faculty of Health Science Research Ethics Board and written informed consent was obtained from each participant including consent to analyze genetic specimens. The investigation conforms to the principles outlined in the Declaration of Helsinki.

### Study Population

The study population was comprised of Canadians of European, South Asian, Chinese, and Aboriginal origin who participated in the Study of Health Assessment and Risk in Ethnic groups (SHARE) and the Study of Health and Research Evaluation in Aboriginal Peoples (SHARE-AP), two cross-sectional studies of cardiovascular disease (CVD) risk factors conducted between 1996 and 1998. Individuals between age 35–75 years were randomly selected from three cities (Toronto, Hamilton, Edmonton), and from the Six Nations Reservation (Ohsweken, Ontario) as previously described [30], [31].

### Clinical Measurements and Biochemical Analyses

We recorded each participant’s health behaviours and medical history using standardized questionnaires as previously described [30], [31]. Blood pressure, height, weight, waist and hip circumference were measured using a standardized protocol [32]. Fasting blood samples were collected in the morning from all participants. Blood samples were collected and processed according to a standard protocol and were shipped to the core laboratory in Hamilton for analysis. All subjects underwent an eight-hour fast before blood was drawn for glucose, insulin, lipids and adiponectin plasma level measurements. Plasma levels of total cholesterol, triglycerides, and glucose were measured using enzymatic methods [33]–[35] and plasma HDL cholesterol level was measured after precipitating the VLDL and LDL with phosphotungstic acid and magnesium chloride [36]. Plasma LDL cholesterol level was calculated according to the method developed by Friedewald et al. [37]. Plasma insulin level was measured by manual radioimmunoassay assay (Diagnostic Products Corporation, Los Angeles USA). Plasma adiponectin level was measured in the laboratory of Dr. Stephan Blankenberg at the University of Mainz, Germany using a commercially available Human adiponectin ELISA assay (RD195023100), produced by Biovendor Research Products. The intra-assay imprecision is 6.4–7.0% and the interassay imprecision is 7.3–8.2%. Basal insulin resistance was calculated using the previously validated HOMA-IR model [38].

### Genetic Analysis

The genotypic information of the rs266729 SNP in *ADIPOQ* was extracted from the gene-centric 50 K single nucleotide polymorphism (SNP) array described elsewhere [39]. Genomic DNA was extracted from leukocytes using the Illumina Human CVD beadchip scanned on the Illumina BeadStation 500 G at the Centre for Applied Genomics for SHARE and from whole blood using the Puregene DNA purification kit (Gentra Systems, Minneapolis MN, USA) at the Clinical Trials and Clinical Research Laboratory (CTCRL) for SHARE-AP. Genotyping of the gene-centric 50 K array was carried out in the Centre for Applied Genomics (Hospital for Sick Children, Toronto, Ontario, Canada; www.tcag.ca) for SHARE and in the Genome Quebec Innovation Center (McGill University, Montreal, QC; www.genomequebec.com) for SHARE-AP. In selecting SNP(s) for analysis, we considered functional rather than surrogate SNPs, since the linkage disequilibrium structure in participants may vary according to the ethnic background. The only available functional SNP in *ADIPOQ* in the gene-centric 50 K array was rs266729. The rs266729 SNP was in Hardy-Weinberg equilibrium within each ethnic group and the call rate for the rs266729 SNP was 98.9%. The polymorphism is common in each ethnic group (G minor allele frequency comprised between 22 and 29% in the four ethnic groups). The G variant is significantly less frequent in Chinese compared to other ethnic groups (**Table S1**).

We also searched for SNPs for use in a reciprocal Mendelian randomization approach [40] to help further decipher the causal relationships between adiponectin level and HOMA-IR. We first searched for a biologically relevant genetic variant conclusively associated with HOMA-IR in the literature and genotyped in the 50 K array. We identified the rs1260326 SNP in *GCKR* as a relevant SNP for this approach. The P446L coding non-synonymous SNP rs1260326 is biologically relevant [41], [42] and has been conclusively associated with HOMA-IR in a French population (P<5×10−8) [43]. The rs1260326 SNP was in Hardy-Weinberg equilibrium within each ethnic group and the call rate for the rs1260326 SNP was 98.9%. The polymorphism is common in each ethnic group (A minor allele frequency comprised between 5% and 21% in the four ethnic groups). The A variant is significantly less frequent in South Asians (5%) and Aboriginals (6%) compared to other ethnic groups (16% in Europeans and 21% in Chinese).

### Statistical Analysis

The statistical analyses were conducted using SAS version 9.1 (Cary, South Carolina), the R package, or PLINK software [44]. Two-tailed P-values <0.05 after Bonferroni correction were considered statistically significant. Hardy-Weinberg equilibrium for each SNP within ethnic groups was assessed by square goodness-of-fit test (P>0.001). The chi-square contingency test was used to compare allele and genotype frequency distributions between ethnic groups and sexes. Non-normally distributed variables were natural log-transformed prior to statistical analyses. Multiple linear regressions were used to assess associations of the rs266729 SNP with quantitative traits, while adjusting for covariates of age, sex and ethnicity coded as indicator variables.

To estimate the genetic effect on metabolic traits as a function of genetically lowered adiponectin levels, we performed a Mendelian randomization analysis. First, we used an instrumental variable analysis in which we determined the quantitative trait change per 1 SD of higher genetically predicted adiponectin concentration. We fit the data to a generalized least squares regression model with the rs266729 SNP as a predictor variable, adjusting for covariates (age, sex, ethnicity), and then we regressed the genetically predicted adiponectin values against the metabolic trait. Second, we calculated a predicted increase in the metabolic parameter based on the effect on adiponectin levels. We converted log adiponectin and log HOMA-IR into z-scores and then used linear regression to model the effect of the rs266729 SNP on log adiponectin z-scores, adjusting for age, sex, and ethnicity. This slope value was then multiplied by the amount that log HOMA-IR z-score changes for every 1 z-score change in log adiopnectin z-score (as determined from a linear regression equation of log adiponectin predicting log HOMA-IR, adjusting for covariates). The resulting value was the predicted increase in the metabolic parameter based on the effect on adiponectin. Third, linear regression was used to assess the effect on log HOMA-IR z-score for the modeled allele. We compared this observed effect with the predicted effect to test whether HOMA-IR may be causally related to rs266729 SNP determined higher adiponectin level. Finally we used a reciprocal Mendelian randomization approach [40] to help further decipher the causal relationships between adiponectin level and HOMA-IR. The functional rs1260326 SNP in *GCKR* is a relevant SNP for a bi-directional Mendelian randomization analysis.

An additive model was used in all the analyses including the rs266729 and the rs1260326 SNP. All of the results, with the exception of the socio-demographic data, are reported based on multivariable analyses. In a sub-analysis, we assessed the association of the metabolic traits with the rs266729 SNP by ethnicity. To further assess the possible heterogeneity in the effects by ethnicity, we conducted a meta-analysis of the parameter estimates obtained for each ethnic group using a random effects model. With a sample size of 1,157, we had 80% power to detect a standard deviation difference in log adiponectin of 0.17 (equal to a difference of 0.12 log ug/mL) and a standard deviation difference in log HOMA-IR as small as 0.17 (absolute difference in log HOMA-IR of 0.105) for a MAF of 20%.

## Results

### Participant Characteristics

Genotype and phenotype data were available from 1,186 participants from the SHARE and SHARE-AP population. From this sample, 29 individuals who had implausible levels of adiponectin (>100 µg/mL) were excluded, leaving a final sample of 1,157 participants (286 Aboriginals, 258 Europeans, 319 South Asians, and 294 Chinese).

The characteristics of participants are displayed in **Table S2**. The mean age of the overall population is 50.3 years. Women are less represented among the South Asians compared to Aboriginals (P = 0.02). Significant differences in age, health behaviours including current smoking, measures of adiposity, glycemic index and load, adiponectin and leptin concentration, and insulin resistance are present among ethnic groups (**Table S2**). For example, Aboriginal people have substantially higher BMI and abdominal obesity relative to other ethnic groups. Conversely people of Chinese origin have the lowest BMI and abdominal obesity. Europeans have a higher BMI, yet less abdominal obesity compared to South Asians. Despite these differences, South Asians and Aboriginal people are more insulin resistant compared to the Europeans (all P<0.05). Adiponectin levels are significantly higher in Europeans and Aboriginals compared to the other ethnic groups (overall P<0.001). (**Table S2**).

### Epidemiologic Assessment of Adiponectin Level and Metabolic Parameters

To investigate the association between adiponectin level and metabolic parameters in this multiethnic sample, multivariable linear regression analyses were performed, adjusting for age, sex and ethnic origin. As shown in **Table 1**, adiponectin level is positively associated with HDL cholesterol (standardized B coefficient = +0.322; P<0.0001) and negatively associated with body mass index (B = –0.193; P<0.0001), waist-to-hip ratio (B = –0.196; P<0.0001), triglycerides (B = –0.247; P<0.0001), fasting glucose level (B = –0.158; P<0.0001), fasting insulin level (B = –0.297; P<0.0001), HOMA-IR (B = –0.297; P<0.0001), systolic (B = –0.098; P = 0.002) and diastolic (B = –0.123; P<0.0001) blood pressure. LDL cholesterol is not associated with adiponectin level (P = 0.42) (**Table 1**). All of the associations remained statistically significant after Bonferoni adjustment for multiple testing (10 tests, P adjusted <0.005).

**Table 1:** Regression coefficients for the relationship between metabolic characteristics and log adiponectin.

	Univariate		Multivariate †	
	Standardizedcoefficient ‡	P-value *	Standardizedcoefficient ‡	P-value *
Body mass index, kg/m^2^	−0.144	<0.0001	−0.193	<0.0001
Waist-to-hip ratio	−0.231	<0.0001	−0.196	<0.0001
LDL cholesterol, mmol/L	−0.059	0.050	−0.024	0.421
Log HDL cholesterol, mmol/L	0.377	<0.0001	0.322	<0.0001
Log triglycerides, mmol/L	−0.270	<0.0001	−0.247	<0.0001
Log glucose, mmol/L	−0.143	<0.0001	−0.158	<0.0001
Log insulin, pmol/L	−0.297	<0.0001	−0.297	<0.0001
Log HOMA-IR	−0.285	<0.0001	−0.297	<0.0001
Systolic blood pressure, mm Hg	−0.086	0.003	−0.098	0.002
Diastolic blood pressure, mm Hg	−0.197	<0.0001	−0.123	<0.0001

† Multivariable linear regression models adjusted for age, sex and ethnicity.

‡ Z scores have a mean of 0 and a SD of 1, the beta coefficient represents the change in log adiponectin level for an increase in 1 SD in the independent variable.

* The p-values for all of the models (except for LDL cholesterol) remain statistically significant after Bonferoni correction.

### Association of rs266729 SNP with Adiponectin Level and Related Metabolic Parameters

We assessed the association of the rs266729 polymorphism, under an additive model, with adiponectin level and metabolic parameters found to be associated with adiponectin in our study. The SNP is significantly associated with serum adiponectin level (**Table 2**). The minor G allele is associated with lower mean adiponectin concentration (G/G: 2.16, C/G: 2.32 and C/C: 2.38 mmol/L log units, respectively) (P = 0.004), adjusting for age, sex, and ethnic origin. Each additional copy of the minor G allele predicts lower adjusted adiponectin by 0.09 mmol/L log units (95% CI: 0.03, 0.15; P = 0.003) (**Table 3**).

**Table 2:** Participant characteristics by ADIPOQ rs266729 genotype. (N = 1157)†.

	CC	CG	GG	
	(N = 621)	(N = 472)	(N = 64)	p-value
Body mass index, kg/m^2^	27.2±0.23	27.4±0.24	27.9±0.65	0.54
Waist-to-hip ratio	0.89±0.004	0.89±0.004	0.90±0.010	0.52
Log adiponectin, µg/mL ‡	2.38±0.020	2.32±0.024	2.16±0.070	**0.004**
LDL cholesterol, mmol/L ‡	3.18±0.03	3.21±0.03	3.22±0.10	0.73
HDL cholesterol, mmol/L ‡ *	1.08±0.01	1.07±0.02	0.95±0.04	**0.004**
Triglycerides, mmol/L ‡ *	1.59±0.06	1.52±0.05	1.67±0.13	0.28
Glucose, mmol/L ‡ *	5.52±0.07	5.55±0.08	5.80±0.22	0.35
Insulin, pmol/L ‡ *	81.0±3.5	83.3±3.5	97.4±7.2	0.08
Log HOMA-IR ‡	1.06±0.023	1.08±0.026	1.28±0.075	**0.003**
Systolic blood pressure, mm Hg ‡	118.7±0.8	118.6±0.8	120.6±2.5	0.70
Diastolic blood pressure, mm Hg ‡	72.5±0.5	72.9±0.5	74.5±1.4	0.40

† Values are mean (SE) or n (%), where applicable.

‡ adjusted for age, sex, and ethnicity.

* geometric mean values.

**Table 3:** Association of rs266729 polymorphism with serum adiponectin, adjusting for covariates.

ADIPOQ SNP (3q27)	Function	Major allele, minor allele (minor allele frequency)	Modeled allele	Effect of modeled allele on serum log adiponectin (µg/mL) †	
				Beta-coefficient (95% CI)	P-value
rs266729	5′-UTR	C, G (0.26)	G	−0.09 (−0.15, −0.03)	0.003

† adjusted for age, sex and ethnicity.

The rs266729 polymorphism is also significantly associated with HDL cholesterol and HOMA-IR, after adjusting for age, sex, and ethnic origin (**Table 2**). The minor allele (G) is associated with lower mean HDL concentration (G/G: 0.95 mmol/L, C/G: 1.07 and C/C: 1.08 mmol/L, respectively) (P = 0.004). The G allele is also associated with higher mean HOMA-IR (C/G and C/C) (G/G: 1.28, C/G: 1.08 and C/C: 1.06 log units, respectively) (P = 0.003). The associations remain statistically significant after accounting for multiple testing (11 tests, P adjusted <0.0045).

All other metabolic characteristics which are associated with adiponectin concentration (body mass index, waist-to-hip ratio, fasting triglycerides, glucose, insulin, and systolic and diastolic blood pressure) are not significantly associated with the rs266729 polymorphism.

The association between the SNP rs266729 and HOMA-IR [45], [46], has been reported previously. Conversely, since the association between the SNP rs266729 and HDL cholesterol was novel, we sought replication of this finding in a publically available independent sample of >100,000 subjects of European ancestry [47]. As the association was not confirmed in this large sample (P = 0.87), we considered the initial significant association between the SNP rs266729 and HDL cholesterol as a random false-positive result, and we did not assess the causal association of adiponectin level and HDL cholesterol in the Mendelian randomization analysis.

### Mendelian Randomization Study for Serum Adiponectin Using SNP rs266729

In order to examine whether a higher HOMA-IR (as observed in carriers of the G allele) may be causally related to the rs266729 SNP determined lower adiponectin level, we performed a Mendelian randomization analysis. If serum adiponectin is causally related to increased insulin resistance, then we would expect that genetically reduced adiponectin (e.g., in individuals carrying the rs266729 G allele) would increase insulin resistance to the same extent as observed in epidemiological studies. We present three main results: i) an estimated causal change in HOMA-IR for a genetically induced change in adiponectin concentration (instrumental variable analysis); ii) a predicted increase in HOMA-IR based on the effect on adiponectin levels (column 6 in **Table 4**); and iii) an observed increase in HOMA-IR (column 7 in **Table 4**).(see Methods section).

**Table 4:** Association of HOMA-IR with the rs266729 polymorphism.

ADIPOQ SNP (3q27)	Function	Major allele, minor allele (minor allele frequency)	Modeled allele	Effect of modeled allele on log serum adiponectin (in SD units) † *	Predicted effect on log HOMA-IR based on effect on log adiponectin † ‡ *	Observed effect on log HOMA-IR for the modeled allele † *	
						Beta-coefficient (95% CI)	P-value
rs266729	5′-UTR	C, G (0.26)	G	−0.14 (−0.24, −0.05)	0.038	0.15 (0.06, 0.24)	<0.001

† , log adiponectin and log HOMA-IR are converted to z-scores.

‡ , this is the amount that log HOMA-IR changes per amount of change in log adiponectin as predicted by the modeled allele in column 5. To determine this predicted effect, we first calculated the amount that log HOMA-IR z-score changes for every 1 z-score change in log adiponectin z-score (as determined from a linear regression equation of log adiponectin predicting log HOMA-IR, adjusting for age, sex, and ethnicity). This value was –0.274 which means that there is a 0.27 SD unit change in HOMA-IR per SD change in adiponectin. This value was then multiplied by column 5 which is simply the effect of the rs266729 SNP modelled allele on log adiponectin z-scores, adjusting for age, sex, and ethnicity.

* adjusted for age, sex, and ethnicity.

**Figure 1:**
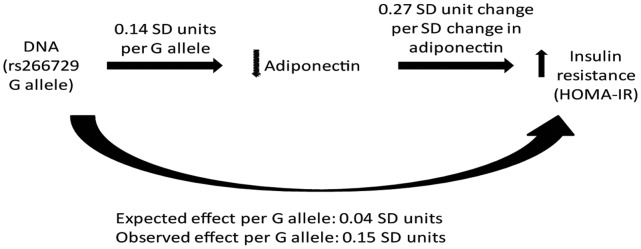
Pictorial summary of the effect of the rs266729 polymorphism on HOMA-IR.

Using instrumental variable analyses, we estimated a causal change in log HOMA-IR of –0.61 (95% CI: –0.70, –0.52; p<0.001) z-score units for a genetically induced change of one log adiponectin z-score unit. Table 4 shows the association of HOMA-IR with the rs266729 SNP. The relationship is also shown pictorially in **Figure 1**. The minor G allele that correlates with lower adponectin is also associated with increased insulin resistance (P<0.001, column 7 in **Table 4**). For this SNP, the observed effect exceeds the effect predicted based on the serum adiponectin change. Specifically, the G allele decreases log adiponectin by 0.14 SD units (95% CI: 0.05, 0.24; P = 0.002) and is expected to increase log HOMA-IR by 0.038 SD units; however we observed a 0.15 SD unit (95% CI: 0.06, 0.24; P<0.001) increase in HOMA-IR. The 95% CI for the estimated causal effect does not include the point estimate for the observed association between adiponectin and HOMA-IR, suggesting that the two estimates are truly different. In order to strengthen confidence in our observation that rs266729 may be causally related to HOMA-IR we reciprocally assessed another biologically relevant SNP known to be associated with HOMA-IR. The SNP rs1260326 in *GCKR* was strongly associated with HOMA-IR (P<0.001), but not with adiponectin level (P = 0.67). Consequently, we did not further explore reciprocal Mendelian randomization analyses using this SNP.

To examine whether the effect of the rs266729 SNP on HOMA-IR is mediated by the variation of adiponectin level, we added log adiponectin to the multivariable model shown in Column 7 of **Table 4**. With the addition of serum adiponectin into the model, the association of the SNP rs266729 with log HOMA-IR (B = 0.09; 95% CI: −0.01, 0.18; P = 0.10) is attenuated and no longer statistically significant.

There is no significant heterogeneity in the gene effect on adiponectin or HOMA-IR variation across ethnic groups (P for interaction = 0.52, and 0.71 respectively) (**Table S3**). To further test if the SNP effects are influenced by ethnicity, we conducted a meta-analysis of the parameter estimates obtained for each ethnic group. The association of adiponectin, HOMA-IR) and the rs266729 SNP were unaltered (**Table S4**).

## Discussion

By using both observational, genetic epidemiology and Mendelian randomization approaches in the multi-ethnic SHARE/SHARE-AP study we found that the adipocyte-secreted hormone adiponectin may have a causal role in the variation of insulin resistance (as measured by the HOMA-IR index). This study is the first to our knowledge to explore the relationships between adiponectin and metabolic traits using Mendelian randomization. Mendelian randomization is thought to overcome the limitations of classical observational epidemiology and provides useful evidence to support or reject causal hypotheses linking intermediary biomarkers and complex diseases [48]. Our findings suggest a possible causal relation between a low serum adiponectin level and a higher level of insulin resistance. More specifically, we show that 1) serum adiponectin level is negatively associated with HOMA-IR, 2) the adiponectin-increasing C allele of the promoter *ADIPOQ* SNP rs266729 is significantly associated with lower HOMA-IR index, 3) the association of the rs266729 SNP with HOMA-IR is attenuated after adjusting for serum adiponectin level in the regression model, 4) the rs266729 SNP is associated with HOMA-IR to an extent that exceeds its effect on adiponectin level, and 5) the validity of this observation is strengthened because the functional SNP rs1260326 in *GCKR* while strongly associated with HOMA-IR, is not associated with adiponectin. Thus our findings do not support a reciprocal causal association of HOMA-IR on adiponectin level.

A strong body of evidence in the literature supports the view that adiponectin may have a causal role in insulin resistance. Serum adiponectin level has been associated with insulin resistance in cross-sectional and longitudinal studies conducted in European [10], [49] and non-European populations [9], [50]–[52]. A recent family-based study showed a heritability (the proportion of total phenotypic variability caused by genetic variance in a population) of 55% for plasma adiponectin and genetic correlations ranging from 18.6 to 20% between plasma adiponectin, HOMA-IR and plasma insulin [15]. These data suggest that the genetic architecture of plasma adiponectin overlaps with the genetics of insulin resistance. Disruption of adiponectin causes a phenotype of insulin resistance in mice [53] and over-expression of adiponectin improves insulin sensitivity wild-type and leptin deficient obese mice [54]–[57]. The physiological mechanisms supporting a link between adiponectin and insulin sensitivity include increased glucose utilization and fatty-acid oxidation in skeletal muscle, suppression of glucose production in the liver, increased storage of triglyceride in the adipocyte and increased synthesis and turn-over of triglycerides in brown adipose tissue [2], [3], [56]–[59]. The significant associations we found between the promoter *ADIPOQ* SNP rs266729, serum adiponectin level and insulin resistance are in line with previous reports [18], [27], [28], [45], [46], [60]–[62]. It is noteworthy that the SNP rs266729 effect on insulin resistance significantly exceeds its predicted effect in the Mendelian randomization experiment. This result is further supported by our finding that adiponectin attenuates rather than negates the effect of the SNP rs266729 on insulin resistance. These data suggest that a low level of adiponectin may alter insulin sensitivity via direct and indirect physiological mechanisms. For instance, insulin resistance state may have effects on gene expression- i.e. negative or positive feedback and amplify the effect of the SNP rs266729 on insulin resistance. Further mechanistic studies are needed to understand the complex relationships between the hormone adiponectin and insulin resistance.

We found no evidence for an interaction between the *ADIPOQ* SNP rs266729 and ethnicity on variation of adiponectin or HOMA-IR. Most of the SNPs identified through GWAS are thought to be proxies in linkage disequilibrium with causal variants and may therefore not been informative in diverse ethnic populations [20], [63], [64]. As an illustration, Croteau-Chonka et al. recently demonstrated that a “synthetic” GWAS association for serum adiponectin level previously reported in the Filipino population [23] was induced by a rare coding variant (R221S) at the *ADIPOQ* locus in linkage disequilibrium with the common SNPs. The R221S variant was unique to the Filipino population and was not found in sequences of 12,514 individuals of European ancestry [20]. Given the similar pattern of genetic effects identified across ethnic groups, our findings illustrate the relevance of using functional instead of tag SNPs in assessing the role of gene variants on metabolic phenotypes in a multi-ethnic Mendelian randomization context [48]. It should be noted that the power of our study remains modest to investigate interactions between the SNP and ethnicity in relation to metabolic traits and further work with this SNP is needed in larger multiethnic samples.

The data suggest that a consistent association between adiponectin and insulin sensitivity is observed in populations of European, South Asian, East Asian or Native Canadian ancestries. This result is in line with previous reports in SHARE/SHARE-AP and in other populations, since they did not provide consistent evidence of varying associations between adiponectin and metabolic traits like insulin resistance according to the ethnic background [65]–[67]. This study is the first to our knowledge to apply Mendelian randomization in a multi-ethnic sample, an important prerequisite to evidence valid causal associations representative of the worldwide human diversity. An important limitation of using Mendelian randomization in a multi-ethnic context therefore resides in its sensitivity to population stratification [48]. We therefore recommend including genetic markers that are robust to inter-ethnic MAF variations in multi-ethnic Mendelian randomization experiments.

Despite the fact that we found a significant association between adiponectin level and HDL cholesterol, HOMA-IR, BMI, WHR, TG, fasting glucose level, fasting insulin level, HOMA-IR, systolic and diastolic blood pressure in our multi-ethnic observational study, we only found a convincing genetic association between the *ADIPOQ* promoter SNP rs266729 and HOMA-IR. One possible explanation is that the association found between adiponectin level and metabolic traits is not causal and explained by confounding factors for all traits but HOMA-IR. However, the modest sample size of the current study (N = 1,157) leads us to not totally exclude the presence of subtle genetic effects of the SNP rs266729 on other metabolic traits that we were unable to detect. For instance, the absence of association between the SNP rs266729 and BMI may be unexpected, since insulin resistance is a key feature of obesity [68]. However, the SNP rs266729 was not associated with BMI in the large-size GIANT study (N >120,000 European subjects, P = 0.30), in line with our results [69]. Another limitation of this study is we have used total adiponectin as opposed to the active high–molecular weight multimer to estimate the circulating levels of adiponectin. Despite the fact that total adiponectin is strongly associated with the HOMA-IR in our study, this may introduce bias the Mendelian randomization model. We are also aware that insulin resistance may have been more precisely estimated using oral glucose tolerance test-derived indexes.

### Conclusion

In conclusion, our data suggest a possible causal association between serum adiponectin level and insulin resistance as measured by HOMA-IR, irrespective of the ethnic background. More Mendelian randomization experiments are needed in well-powered multi-ethnic studies to decipher the complex pleiotropic role of the adipocyte-secreted hormone adiponectin on metabolic traits.

## Supporting Information

Table S1 **Characteristics of the rs266729 SNP genotyped in Aboriginal, South Asian, Chinese and European participants.**
(DOC)

Table S2 **Characteristics of study participants by ethnicity.**
(DOC)

Table S3 **Association of metabolic traits with the rs266729 polymorphism. Values are beta-coefficient (95% CI). †**
(DOC)

Table S4 **Association of metabolic traits with the rs266729 polymorphism, based on meta-analysis of adjusted estimates obtained for each ethnic group. Values are based on fixed effect model. †**
(DOC)
