# Supplementary material for: Causal Relationship between Adiponectin and Metabolic Traits: A Mendelian Randomization Study in a Multiethnic Population
Source: PLoS One. 2013 Jun 24;8(6):e66808. doi: 10.1371/journal.pone.0066808 (PMC3691277; doi:10.1371/journal.pone.0066808)
Supplement: Table S1 — Characteristics of the rs266729 SNP genotyped in Aboriginal, South Asian, Chinese and European participants. (DOC) [file pone.0066808.s001.doc]

| **Supplementary Table 1**. **Characteristics of the rs266729 SNP genotyped in Aboriginal, South Asian, Chinese and European participants.** | | | | | | | | | | | | | |
| --- | --- | --- | --- | --- | --- | --- | --- | --- | --- | --- | --- | --- | --- |
| Major allele, minor allele (minor allele frequency) | Genotype frequency † | | | | | | | | | | | | Overall P-value |
|  | Aboriginal | | | South Asian | | | Chinese | | | European | | |  |
|  | CC | CG | GG | CC | CG | GG | CC | CG | GG | CC | CG | GG |  |
| C, G (0.26) | 150 (52.4) | 120 (42.0) | 16 (5.6) | 160 (50.2) | 136 (42.6) | 23 (7.2) | 178 (60.5) | 104 (35.4) | 12 (4.1) | 133 (51.6) | 112 (43.4) | 13 (5.0) | 0.002 ^1-3^ |
| Call rate | 99.0% | | | 98.8% | | | 99.9% | | | 98.5% | | |  |
| Minor allele frequency | 0.27 | | | 0.29 | | | 0.22 | | | 0.27 | | |  |
| Hardy-Weinberg, p-value | 0.20 | | | 0.42 | | | 0.51 | | | 0.08 | | |  |

† Chi-square test for genotype frequency by ethnic group is p=0.17.

1 P<0.05, Chinese versus European.

2 P<0.05, Chinese versus South Asian.

3 P<0.05, Chinese versus Aboriginal.
