# Supplementary material for: Causal Relationship between Adiponectin and Metabolic Traits: A Mendelian Randomization Study in a Multiethnic Population
Source: PLoS One. 2013 Jun 24;8(6):e66808. doi: 10.1371/journal.pone.0066808 (PMC3691277; doi:10.1371/journal.pone.0066808)
Supplement: Table S2 — Characteristics of study participants by ethnicity. (DOC) [file pone.0066808.s002.doc]

| **Supplementary Table 2. Characteristics of study participants by ethnicity.** | | | | | | |
| --- | --- | --- | --- | --- | --- | --- |
|  | Total (N=1,158) | Aboriginal (N=279) | European (N=262) | South Asian (N=320) | Chinese (N=297) | Overall P-value |
| Age, years | 50.3 ± 0.3 | 53.0 ± 0.6 | 51.3 ± 0.6 | 49.5 ± 0.6 | 47.7 ± 0.6 | <0.001 ^1-6^ |
| Female, N (%) | 591 (51.0) | 161 (57.7) | 139 (52.9) | 146 (45.5) | 145 (48.5) | 0.02 ^5,6^ |
| Current smoker, N (%) | 199 (17.1) | 111 (39.8) | 39 (14.9) | 32 (10.0) | 17 (5.7) | <0.001 ^1,3,5,6^ |
| Body mass index, kg/m^2^ † | 27.2 ± 0.2 | 31.9 ± 0.3 | 27.5 ± 0.3 | 26.2 ± 0.3 | 23.9 ± 0.3 | <0.001 ^1-6^ |
| Waist-to-hip ratio † | 0.89 ± 0.003 | 0.94 ± 0.004 | 0.87 ± 0.004 | 0.88 ± 0.003 | 0.86 ± 0.004 | <0.001 ^2-6^ |
| Glycemic index † | 151 ± 2.0 | 178 ± 4.3 | 156 ± 4.0 | 151 ± 3.9 | 122 ± 3.9 | <0.001 ^1,3-6^ |
| Glycemic load † | 57 ± 0.2 | 61 ± 0.3 | 56 ± 0.3 | 57 ± 0.2 | 53 ± 0.2 | <0.001 ^1-6^ |
| Adiponectin, μg/mL ‡ | 10.38 ± 0.4 | 10.59 ± 0.5 | 12.18 ± 0.7 | 9.97 ± 0.5 | 9.30 ± 0.6 | <0.001 ^1-3,5^ |
| HOMA-IR ‡ | 2.94 ± 0.2 | 4.22 ± 0.3 | 2.27 ± 0.1 | 3.10 ± 0.2 | 2.48 ± 0.1 | <0.001 ^2-6^ |

† Means are adjusted for age and sex.

‡ Geometric means are adjusted for age, sex and waist-to-hip ratio.

1 P<0.05, European versus Chinese.

2 P<0.05, European versus South Asian.

3 P<0.05, European versus Aboriginal.

4 P<0.05, Chinese versus South Asian.

5 P<0.05, Chinese versus Aboriginal.

6 P<0.05, South Asian versus Aboriginal.
