# Supplementary material for: Causal Relationship between Adiponectin and Metabolic Traits: A Mendelian Randomization Study in a Multiethnic Population
Source: PLoS One. 2013 Jun 24;8(6):e66808. doi: 10.1371/journal.pone.0066808 (PMC3691277; doi:10.1371/journal.pone.0066808)
Supplement: Table S3 — Association of metabolic traits with the rs266729 polymorphism. Values are beta-coefficient (95% CI). † (DOC) [file pone.0066808.s003.doc]

| **Supplementary Table 3. Association of metabolic traits with the rs266729 polymorphism. Values are beta-coefficient (95% CI).** **†** | | | | | |
| --- | --- | --- | --- | --- | --- |
|  | Total (N=1,158) | Aboriginal (N=279) | European (N=262) | South Asian (N=320) | Chinese (N=297) |
| Adiponectin ‡ | -0.14 (-0.24, -0.05) * | -0.22 (-0.36, -0.07) * | -0.17 (-0.39, 0.05) | -0.06 (-0.23, 0.12) | -0.18 (-0.38, 0.02) |
| HDL-C ‡ | -0.14 (-0.24, -0.05) * | -0.07 (-0.24, 0.10) | -0.03 (-0.21, 0.15) | -0.22 (-0.39, -0.05) * | -0.06 (-0.24, 0.12) |
| HOMA-IR ‡ | 0.15 (0.06, 0.24) * | 0.07 (-0.11, 0.25) | 0.14 (-0.01, 0.29) | 0.15 (0.01, 0.29) * | 0.13 (-0.02, 0.28) |

† Beta-coefficients are adjusted for age, sex and ethnicity.

‡ Log adiponectin, log HDL, and log HOMA-IR are converted to z-scores.

* Statistically significant.
