# Supplementary material for: Causal Relationship between Adiponectin and Metabolic Traits: A Mendelian Randomization Study in a Multiethnic Population
Source: PLoS One. 2013 Jun 24;8(6):e66808. doi: 10.1371/journal.pone.0066808 (PMC3691277; doi:10.1371/journal.pone.0066808)
Supplement: Table S4 — Association of metabolic traits with the rs266729 polymorphism, based on meta-analysis of adjusted estimates obtained for each ethnic group. Values are based on fixed effect model. † (DOC) [file pone.0066808.s004.doc]

| **Supplementary Table 4. Association of metabolic traits with the rs266729 polymorphism, based on meta-analysis of adjusted estimates obtained for each ethnic group. Values are based on fixed effect model.** **†** | | | |
| --- | --- | --- | --- |
|  | **Summary beta-coefficient (95% CI),** | **P-value** | **P-value for test of heterogeneity** |
| Adiponectin ‡ | -0.16 (-0.25, -0.07) * | <0.001 | 0.61 |
| HDL-C ‡ | -0.10 (-0.19, -0.01) * | 0.03 | 0.42 |
| HOMA-IR ‡ | 0.13 (0.05, 0.20) * | <0.0001 | 0.90 |

† Beta-coefficients are adjusted for age and sex.

‡ Log adiponectin, log HDL, and log HOMA-IR are converted to z-scores.

* Statistically significant.
